# Phase-Field Crystal Method for Bilayer Graphene

**DOI:** 10.3390/nano15221699

**Published:** 2025-11-10

**Authors:** Heting Qiao, Kai Liu

**Affiliations:** 1 School of Mechanical Engineering, Inner Mongolia University of Technology, Hohhot 010051, China; qiaoheting311@163.com; 2 Faculty of arts and sciences, Beijing Normal University at Zhuhai, No.18 Jinfeng Road, Zhuhai 519087, China

**Keywords:** bilayer graphene, generalized stacking fault energy, phase-field method

## Abstract

Bilayer graphene has been a subject of intense study in recent years. Its most common structure is AB (Bernal) stacking, where one layer is shifted relative to the other, leading to distinct electronic behaviors compared to the less stable AA stacking or the fascinating twisted configurations. We extend a structural phase field crystal method to include an external potential based on the generalized stacking-fault energy that accounts for the effect from a bottom layer of graphene. Both of the favored stacking variants AB and BA are found with randomly generated initial phase fields. Using the width of the boundaries between different stacking variants as a function of the interactions between the two layers, we quantify the exact strength of the external potential by comparing the phase field crystal simulations with the results from atomistic simulation. Finally, we simulate a circular grain of one stacking phase enclosed by the other and find that, depending on the initial phase field, the center domain may shrink to form a uniform stacking phase, or may evolve to a relaxed state of a hexagon region or a triangular region that at each vertex the graphene structure is defected.

## 1. Introduction

Graphene, a single layer of carbon atoms tightly bound in a hexagonal honeycomb lattice, is one of the most exciting two-dimensional materials discovered. Bilayer graphene, consisting of two coupled, single layers with a honeycomb carbon crystal structure, has attracted a great deal of attention because it can exist with a variety of stacking arrangements with intriguing electronic properties [1,2,3]. Unlike monolayer graphene, which possesses a zero bandgap, the electronic structure of bilayer graphene can be significantly modified by external fields, mechanical distortions, and structural variations, enabling a wide range of electronic phenomena from tunable semiconductors to unconventional superconductivity [4,5,6]. Its most common structure is AB (Bernal) stacking, where one layer is shifted relative to the other, leading to distinct electronic behaviors compared to the less stable AA stacking or the fascinating twisted configurations [7,8]. The growing sophistication in synthesis, particularly the controlled growth of twisted bilayers, coupled with a deeper understanding of the role of defects and interactions, promises to unlock further potential [9,10,11].

Computational modeling can serve as a route for theoretically understanding the difficult-to-measure properties of graphene. On the continuum scale, the phase-field crystal (PFC) modeling approach describes the thermodynamics and dynamics of phase transformation through an atomically varying order parameter field that is loosely connected to the atomic density field. It is a special type of phase-field model which enables the order parameter to remain stable in both uniform and periodic states, where the periodic state represents the coarsened atomic structure of the crystalline phase on the diffusive time scale [12,13,14].

The original PFC model was predominately used for the study of 2D triangular and three-dimensional (3D) crystal symmetries [12,15,16,17,18,19]. PFC simulations make it possible to study solidification processes and elastoplastic material responses, covering a wide range of phenomena. These phenomena include the formation and co-evolution of microstructural defects such as dislocations and stacking faults [20], defect formation in epitaxial growth [21], displacive phase transitions [22], corrosion rate and morphology of a dealloying metal [23], voids [24,25,26], and electromigration [27]. The PFC model possesses both continuum and atomic-scale characteristics, which makes it particularly useful for investigating how atomic-scale structures affect mesoscale properties that govern microstructural evolution. These properties range from interface energy anisotropy [28,29] to sublattice ordering in order-disorder transformations [30] and atomic structures in eutectic growth [31]. By including a rotationally invariant three-point correlation function for the excess free energy, a structural PFC model was setup to address both the atomically varying defect and microstructures of graphene and its nucleation and diffusional growth kinetics from a disordered state on a surface [32]. This method allows for a more systematic and adaptable parameterization of diverse crystal structures. The formalism has been extended to reproduce parts of real material phase diagrams, such as those of aluminum and tungsten, by fitting the model parameters to experimental data [33,34].

In this work, we develop a PFC model for bilayer graphene by extending the structural PFC approach introduced in [32]. Following [32], we employ a structural PFC model that incorporates both two- and three-point correlation kernels in the nonlocal contribution to the free energy. To capture the effect of a second graphene layer on the modeled layer, we introduce a local interaction between the order parameter density and an external potential, referred to as the bottom-layer potential. This approach corresponds to a physical scenario in which the bottom graphene layer is fixed on a substrate, and the layer under consideration is deposited on top of it. The form of the bottom-layer potential is informed by first-principles calculations of the generalized stacking fault energy (GSFE) in bilayer graphene from [35], which describes the energy variation associated with the lateral displacement of one graphene layer relative to the other. The analytic expression of the GSFE provided in [35] implies that a potential of the same functional form—though with different coefficients—would exactly reproduce the GSFE in an atomistic simulation [36]. In our PFC model, we adopt this functional form for the bottom-layer potential, but scale it by an adjustable parameter. The value of this parameter is determined by fitting against molecular dynamics (MD) simulations [36,37], which are themselves validated against experimental results, as discussed later in the text.

In the numerical simulations, we first test the growth of graphene phases without the bottom-layer potential by choosing parameters corresponding to the solid region of the phase-field, i.e., ensuring the periodicity for each side of the rectangular domain and initializing the system with Gaussian noise. The results agree with [32]. We then add bottom-layer potential and again start with Gaussian noise and generate both the AB and BA stacking, where AB and BA stacking have one of the first layer’s sublattice atoms (A or B) directly on top of its opposite sublattice atom (B or A) in the second layer, called AB or BA stacking, respectively, or collectively called Bernal stacking [38]. Then, we test the case of a long, narrow ribbon domain. The initial condition consists of four parts, continuous AB and BA regions each of nearly 50% of the entire domain, respectively, and two narrow transitions between each of them is set to be a constant with small Gaussian perturbation. We find four transition types, depending on the angle between the transition region and the shifting direction, 0°, 30°, 60°, and 90°. By comparing with the simulation results from atomistic methods [39,40], we quantify the strength of the bottom-layer potential by the width of the transition region for each type of transition. Finally, we test the case of a circular AB vs BA stacking. With constant transition region, the center part (even a relatively small one) may evolve to a triangular or hexagonal shape with at least one 5–7 defective ring on each vertex. On the other hand, with uniformly smooth transition from AB to BA, the area of the center part will shrink at a constant speed (even for a relatively large disk).

The simulations are so numerically expensive that they take a long time, i.e., 3–7 days, to reach a steady solidification. In order to solve the system in a large domain efficiently, we use a CUDA C/C++ programming on Nvidia Quadro GV100 (NVIDIA Corporate, Santa Clara, CA, USA) which runs about two orders of magnitude faster than the normal version.

## 2. Modeling and Method

### 2.1. Modeling

We incorporate an external bottom-layer potential into the structural PFC model [32,35]. Let ρ describe the spatial phase density of carbon atoms. A dimensionless density field is then defined as $\psi =\left(\rho -\bar{\rho }\right)/\bar{\rho }$, where $\bar{\rho }$ is the mean value of ρ. The free energy of a crystallizing system reads as

(1) $$F_{\mathrm{total}}=F_{\mathrm{id}}\left(\psi \right)+F_{\mathrm{ex},2}\left(\psi \right)+F_{\mathrm{ex},3}\left(\psi \right)+F_{\mathrm{BL}}\left(x\right),$$

where $F_{\mathrm{id}}$ is the ideal free energy, $F_{\mathrm{ex},2}$ the two-point interactions, $F_{\mathrm{ex},3}$ the three-point correlations [32], and $F_{\mathrm{BL}}\left(x\right)$ the energy of the bottom-layer potential [35].

$F_{\mathrm{id}}$ is given by

(2) $$F_{\mathrm{id}}=\int dx\left\{\frac{\psi^{2}}{2}-\eta \frac{\psi^{3}}{6}+\chi \frac{\psi^{4}}{12}\right\},$$

where η and χ are dimensionless parameters and we simply set η=χ=1. The two-point correlation is based on hard-sphere-like interactions and it is governed by [32]:

(3) $$F_{\mathrm{ex},2}=-\frac{1}{2}\int \psi \left(x\right)\int C_{2}\left(x-x^{\prime }\right)\psi \left(x^{\prime }\right)dx^{\prime }dx.$$

Here, $C_{2}$ is the two-point correlation function defined as [32]:

(4) $$C_{2}\left(x\right)=-\frac{R}{\pi r_{0}^{2}}\mathrm{circ}\left(\frac{r}{r_{0}}\right),$$

where $r_{0}$ sets the cutoff for the repulsive term, *R* sets the magnitude of the repulsion, and

(5) $$\mathrm{circ}\left(r\right)=\left\{\begin{array}{c} 1,\;r\leq 1,\\ 0,\;r>1. \end{array}\right.$$

The three-point density correlation, which can describe the crystallography of structurally more complex phases than 2D triangulars in a unified way, is rotationally invariant and robust enough to capture all crystal structures described through a single bond angle [32]. The three-point correlations is governed by

(6) $$F_{\mathrm{ex},3}=-\frac{1}{3}\int \psi \left(x\right)\int C_{3}\left(x-x^{\prime },x-x^{\prime \prime }\right)\psi \left(x^{\prime }\right)\psi \left(x^{\prime \prime }\right)dx^{\prime }dx^{\prime \prime }dx.$$

Here, $C_{3}$ is

(7) $$C_{3}\left(x-x^{\prime },x-x^{\prime \prime }\right)=\sum_{i}C_{s}^{\left(i\right)}\left(x-x^{\prime }\right)C_{s}^{\left(i\right)}\left(x-x^{\prime \prime }\right),$$

where $C_{s}^{\left(i\right)}$ in polar coordinate reads as [32]:

(8) $$\begin{array}{cc} C_{s}^{\left(1\right)}\left(r,\theta \right) & =C_{r}\left(r\right)\cos\left(m\theta \right), \end{array}$$

(9) $$\begin{array}{cc} C_{s}^{\left(2\right)}\left(r,\theta \right) & =C_{r}\left(r\right)\sin\left(m\theta \right), \end{array}$$

with $C_{r}\left(r\right)=\frac{X}{2\pi a_{0}}\delta \left(r-a_{0}\right)$. Here, *X* is a parameter defining the strength of the interaction, $a_{0}$ corresponds to the lattice spacing and $r_{0}/a_{0}=1.22604$, and m=3 defines bond order of the crystal phase. For the graphene system, R=6 and $X^{-1}=0.4$ [32].

The energy of the bottom-layer potential reads as

(10) $$\begin{array}{cc} F_{\mathrm{BL}}= & \frac{1}{\lambda }\int dx\left[\psi \left(x\right)V_{\mathrm{BL}}\left(x\right)\right], \end{array}$$

(11) $$\begin{array}{cc} V_{\mathrm{BL}}\left(x,y\right)= & c_{0}+c_{1}\left[\cos\frac{2\pi }{a_{s}}\left(x+\frac{y}{\sqrt{3}}\right)+\cos\frac{2\pi }{a_{s}}\left(x-\frac{y}{\sqrt{3}}\right)+\cos\frac{4\pi y}{\sqrt{3}a_{s}}\right]\\ \; & +c_{2}\left[\cos\frac{2\pi }{a_{s}}\left(x+\sqrt{3}y\right)+\cos\frac{2\pi }{a_{s}}\left(x-\sqrt{3}y\right)+\cos\frac{4\pi x}{a_{s}}\right]\\ \; & +c_{3}\left[\cos\frac{2\pi }{a_{s}}\left(2x+\frac{2y}{\sqrt{3}}\right)+\cos\frac{2\pi }{a_{s}}\left(2x-\frac{2y}{\sqrt{3}}\right)+\cos\frac{8\pi y}{\sqrt{3}a_{s}}\right]\\ \; & +c_{4}\left[\sin\frac{2\pi }{a_{s}}\left(x-\frac{y}{\sqrt{3}}\right)-\sin\frac{2\pi }{a_{s}}\left(x+\frac{y}{\sqrt{3}}\right)+\sin\frac{4\pi y}{\sqrt{3}a_{s}}\right]\\ \; & +c_{5}\left[\sin\frac{2\pi }{a_{s}}\left(2x-\frac{2y}{\sqrt{3}}\right)-\sin\frac{2\pi }{a_{s}}\left(2x+\frac{2y}{\sqrt{3}}\right)+\sin\frac{8\pi y}{\sqrt{3}a_{s}}\right], \end{array}$$

where $a_{s}$ is the distance between two nearby minimum points, i.e., the center of two nearby holes on the bottom layer of graphene. $c_{0}=21.336$, $c_{1}=12.254$, $c_{2}=-1.128$, $c_{3}=-0.286$, $c_{4}=\sqrt{3}c_{1},$ and $c_{5}=-\sqrt{3}c_{3}$ [35]. The independent coefficients are given in terms of the coefficients in Table III of ref. [35] by $c_{1,\mathrm{BL}}=-2c_{1,\mathrm{GSFE}}$, $c_{2,\mathrm{BL}}=c_{2,\mathrm{GSFE}},$ and $c_{3,\mathrm{BL}}=-2c_{3,\mathrm{GSFE}},$ where BL indicates our coefficients, and GSFE those from ref. [35]. Because the bottom graphene layer is represented by an external potential, it does not deform, just as if it were fixed by a deposition substrate. Also note that by doing so, the effect from the top layer to the bottom layer is not counted. This is partly because the bottom layer is attached to another surface, that the mechanical response from the top layer is less significant.

**Figure 1:**
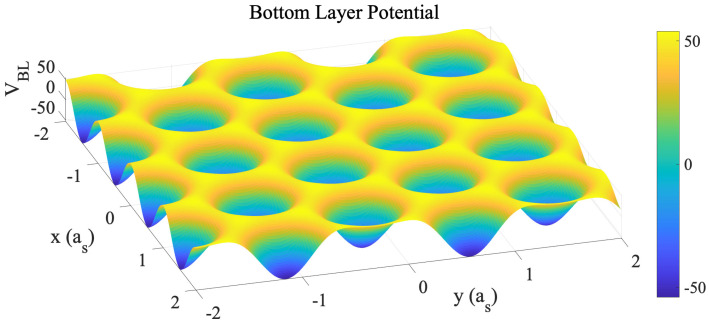
Spatial distribution of the dimensionless bottom-layer potential when λ=1. The maximum and the minimum points are the location of carbon atoms and holes on the bottom layer, correspondingly.

Here, λ is a parameter measuring the strength of the bottom-layer potential. Since the structural PFC method is based on geometric configuration rather than actual physical quantities, the strength of the stacking fault energy between the two graphene layers cannot be directly anchored by corresponding physical parameters. Therefore, an additional parameter must be introduced to determine the actual stacking energy. The bottom-layer potential when λ=1 is shown by Figure 1. Note that it only displays the geometric configuration of the GSFE that corresponds to a honeycomb hexagonal pattern representing the structure of bottom layer, where the maximum and the minimum points are the location of carbon atoms and center of the holes on the bottom layer correspondingly.

Define $a_{c}$ as the lateral distance between two nearby atom centers, that $a_{c}=\sqrt{3}a_{s}/3$ for a well-structured graphene. In the structural PFC model [32], $a_{c}=\sqrt{3}a_{0}/2\pi ;$ therefore, $a_{s}=3a_{0}/2\pi .$ Finally, the evolution of the density ψ, which is a conserved order parameter, is governed by

(12) $$\frac{\partial \psi }{\partial t}=M_{\psi }\nabla^{2}\left(\frac{\delta F_{\mathrm{total}}}{\delta \psi }\right),$$

where $M_{\psi }$ is an effective mobility that scales of the diffusional dynamics of ψ and we set $M_{\psi }=1$ for convenience.

In the molecular dynamics (MD), or more literally, molecular statics, the intralayer potential was the Adaptive Intermolecular Reactive Empirical Bond Order (AIREBO) Potential [39,41] as implemented in LAMMPS [42], and a interlayer potential following [40] was used. The same as the combination of potentials used by [3]. We implemented interlayer potential in LAMMPS, with the $r^{-6}$ term cut off abruptly at 5.0 nm. The simulation cells were 198 to 201 nm normal to the boundary, and 1.2 to 2.2 nm along the boundary, depending on the boundary character. The starting configurations were created by interpolating the displacement in the upper layer linearly over a distance of 6 nm normal to the boundary. During relaxation, the cell length parallel to the boundary is kept fixed, but the length normal to the boundary is relaxed.

**Figure 2:**
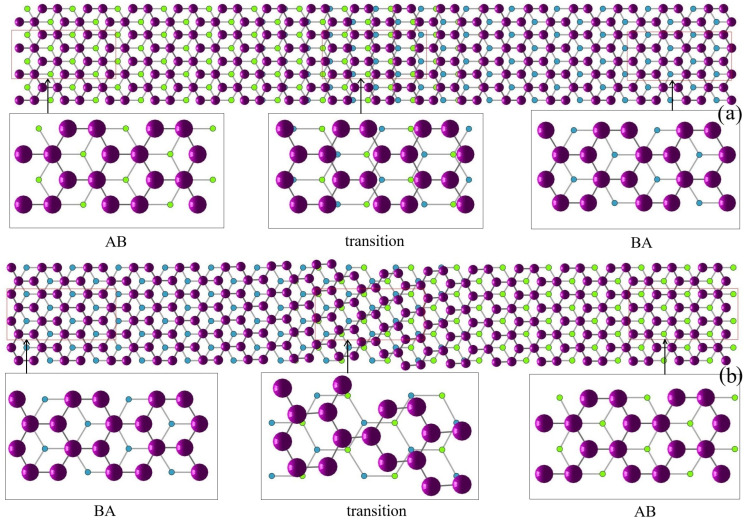
(**a**) The AB to BA transition on the left region, where the atoms shift horizontally, and the transition region is of 90 degree. (**b**) The BA to AB transition on the right region. The transition region is of 30 degree.

For the MD method, simulations were conducted with periodic boundary conditions for both in-plane directions, and two straight boundaries with opposite (partial) burgers vectors. A sketch of the boundary structure is given in Figure 2. The bottom layer was kept flat. Two sets of simulations were conducted. In one, the atoms in the bottom layer were kept fixed at their positions in the perfect graphene crystal (fixed bottom). In the other case, the atoms in the bottom layer could move freely within the plane (flat bottom). For the flat bottom simulations, the two boundaries are essentially equivalent, and their widths are essentially the same. For the fixed bottom simulations, this is not the case, and the two boundaries may have different widths, except for the pure shear case where the boundaries are equivalent. The reason the two boundaries differ in the fixed bottom simulations, when there is any edge component to the burgers vector, is that the bottom layer is kept fixed, and the top layer in one of the boundaries is stretched, and in the other boundary, it is compressed. In the flat boundary, one layer is stretched, and the other compressed for both boundaries. The top layer is still different from the bottom layer because it is not kept flat, but this is not enough to affect the width significantly.

### 2.2. Numerical Methods

#### 2.2.1. FFT Method

We use a discrete Fourier transform (DFT) method to solve Equation (12). The two-point correlation structure $F_{\mathrm{ex},2}$ is computed by [32]:

(13) $$\frac{\partial F_{\mathrm{ex},2}}{\partial \psi }=-\int C_{2}\left(x-x^{\prime }\right)\psi \left(x^{\prime }\right)dx^{\prime }\equiv -C_{2}*\psi ,$$

where ∗ is a convolution in the domain, and in the reciprocal space:

(14) $${\hat{C}}_{2}\left(k\right)=-2R\frac{J_{1}\left(r_{0}k\right)}{r_{0}k},\;k=\sqrt{k_{1}^{2}+k_{2}^{2}},$$

where $J_{m}$ are the Bessel functions of the first kind, and $k_{1}$, $k_{2}$ are the modes in the 2D reciprocal space. The three-point correlation structure $C_{3}\left(r-r^{\prime },r-r^{\prime \prime }\right)$ is separated to

(15) $$C_{3}\left(r-r^{\prime },r-r^{\prime \prime }\right)=\sum_{i}C_{s}^{\left(i\right)}\left(r-r^{\prime }\right)C_{s}^{\left(i\right)}\left(r-r^{\prime \prime }\right).$$

The $F_{\mathrm{ex},3}$ is then computed as [32]:

(16) $$\frac{\partial F_{\mathrm{ex},3}}{\partial \psi }=-\frac{1}{3}\left\{{\left[C_{s}^{\left(i\right)}*\psi \right]}^{2}+2{\left(-1\right)}^{m}C_{s}^{\left(i\right)}*\left[\psi \cdot \left(C_{s}^{\left(i\right)}*\psi \right)\right]\right\},$$

where

(17) $$\begin{array}{cc} {\hat{C}}_{s}^{\left(1\right)}\left(k,\theta_{k}\right) & =Xi^{m}\cos\left(m\theta_{k}\right)J_{m}\left(ka_{0}\right), \end{array}$$

(18) $$\begin{array}{cc} {\hat{C}}_{s}^{\left(2\right)}\left(k,\theta_{k}\right) & =Xi^{m}\sin\left(m\theta_{k}\right)J_{m}\left(ka_{0}\right), \end{array}$$

with

(19) $$k=\sqrt{k_{1}^{2}+k_{2}^{2}},\;\;\theta_{k}=\arctan\left(\frac{k_{2}}{k_{1}}\right).$$

#### 2.2.2. Scaling the Domain to Incorporate the Bottom-Layer Potential

To incorporate $F_{\mathrm{BL}}$, we need to ensure the rectangular domain has the same periodicity as $V_{\mathrm{BL}}\left(x\right)$. Thus, the aspect ratio of the rectangular domain must be $n_{1}:\sqrt{3}n_{2}$, where $n_{1}$ and $n_{2}$ are positive integers. For rectangular domain $L\times \sqrt{3}L$, by setting

(20) $$k=\sqrt{k_{1}^{2}+3\times k_{2}^{2}},\;\;\theta_{k}=\arctan\left(\frac{\sqrt{3}\times k_{2}}{k_{1}}\right),$$

$C_{2}$ and $C_{s}^{\left(i\right)}$ functions are then of circular shapes. Note that the Laplacian operator also needs to be scaled to ensure that the diffusion is invariant of direction.

#### 2.2.3. GPU Acceleration

We solve the system in a large domain, e.g., $N_{X}\times N_{Y}=1024\times \left(32\times 1024\right)$. FFT is extremely efficient by GPU parallelization, since the bandwidth of the memory is the bottleneck of the performance for FFT algorithm by machines with enough computational power, like a cluster with tens of cores or a Nvidia Tesla GPU card with thousands of nodes. Here, we use a CUDA C/C++ parallel programming (based on CUDA Toolkit 11.1.0) on a Linux system (Ubuntu 20.04 LTS), which is fully developed by ourselves, to accelerate our algorithm, which runs about two orders of magnitude faster than normal CPU version (using AMD Ryzen 5700 chip). The Nvidia Quadro GV100 is used, whose memory bandwidth is 868.4 GB/s.

## 3. Results

### 3.1. Preliminary Test of the Model

**Figure 3:**
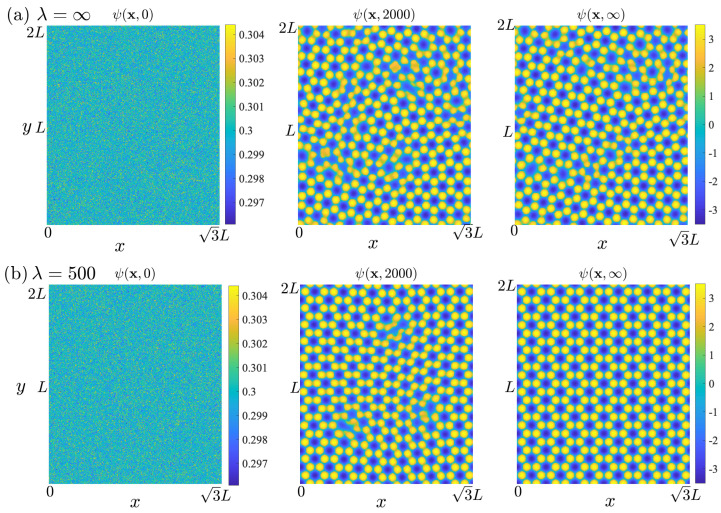
Density fields, ψ(x,t), of the upper graphene layer for (**a**) λ=∞ and (**b**) λ=500, showing the evolution at initial (T=0), early (T=2000), and late (T≥ 20,000) stages of solidification. The color bars are shared between the middle and rightmost panels for each case.

We set the initial phase-field ψ(x,0)=0.3+Z(x), where Z(x) is a spatially uncorrelated scalar field of Gaussian noise with mean 0 and standard deviation 0.001. As shown in Figure 3a, when $F_{\mathrm{BL}}=0$, i.e., λ=∞, the graphene evolves to a defected structure that agrees with [32]. Then, we set λ=500 with the same initial phase-field, that the graphene evolves to a neat structure as shown in Figure 3b, and it takes a shorter time to reach such an equilibrium.

**Figure 4:**
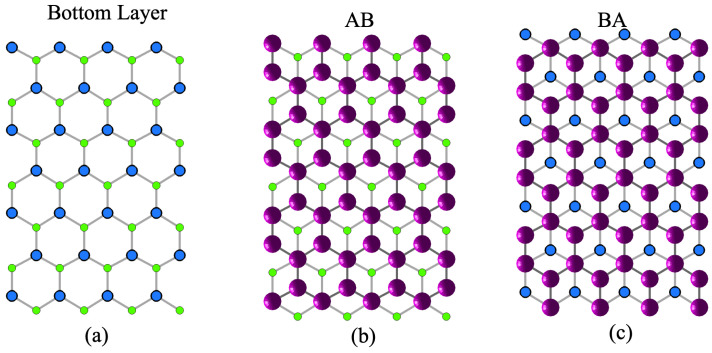
(**a**) Bottom-layer atoms are divided into two groups: one is denoted by lighter green and the other by darker blue. (**b**) Position of atoms for AB stacking order. (**c**) Position of atoms for BA stacking order.

Next, we simulate a group of samples by setting λ=500 and initial phase-field again ψ(x,0)=0.3+Z(x) yet with different random seeds. Both AB and BA stacking phases are found [38], as shown in Figure 4. In order to display the structure clearly, in Figure 4a, we plot the bottom layer the way that the carbon atoms are divided into two groups with one group colored by lighter green and the other by darker blue. One lighter, green atom is near to three darker atoms and vice versa. In Figure 4b, we plot AB stacking order where the upper layer atoms locate above the darker blue atoms and the center of the hexagons, different from BA stacking, as shown by Figure 4c where the upper layer atoms locate right above the lighter green atoms and in the center of the hexagons.

We also find that by adding $F_{\mathrm{BL}}\left(\lambda =500\right)$ to a defected graphene, the phase-field will evolve to a well-structured state, like in Figure 3b.

### 3.2. The Transition Between the AB and BA Stacking Order

**Figure 5:**
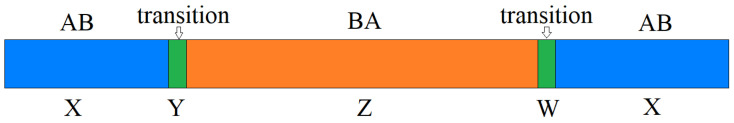
A script of the initial phase-field for a long ribbon with transition between AB and BA.

Given a long ribbon of bilayer graphene, where AB phase and BA phase are of equal length, we investigate the transition between them. The initial state is a static setup where there are four parallel stripes X-Y-Z-W-X, as shown in Figure 5, X represents the stacking order AB, Z represents the stacking order BA, and Y, W are disordered. The X and Z region grows as the dynamics start and two interfaces will be created between them. As shown in Figure 2, there are two boundaries: one from AB to BA and the other from BA to AB.

Here, the angle θ between the direction of the transition region and the direction along which the atoms shift is used as the dislocation character. In Figure 2, for example, the transition region (X-Z-Y) is vertical and the left region is 90 degree (the atoms shift horizontally) as shown in Figure 2a, while the right region (Y-W-X) is 30 (or −30) degree, as shown in Figure 2b.

We then compute the thickness of the transition region. Define a nondimensionalized parameter $d_{L}$ that measures the distortion between substrate potential $V_{\;\mathrm{BL}}\left(x\right)$ and the graphene field ψ(x):

(21) $$d_{L}=\mathrm{mean}\left(d_{c_{i}}\right)$$

where $d_{c_{i}}$ is the nondimensionalized *x*-*y* plane distance between the center of one group of atoms on the bottom layer and the nearest atom centers on the top layer at $x_{i}$, for example, the distance in *x*-*y* plane between the center of the lighter green circles and the center of pink balls in Figure 2. Here, $d_{L}=1$ for AB pattern and $d_{L}=0$ for BA pattern. The data is then fitted by the function

(22) $$d_{L}=\frac{1}{\pi }\arctan\left(\exp\left(\frac{\pi L}{W}\right)\right)+\frac{1}{2},$$

where *W* is a fitting parameter that stands for the thickness of the transition region.

**Figure 6:**
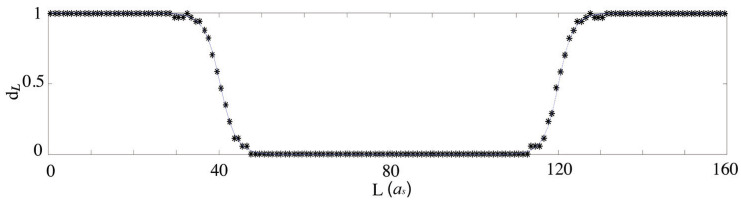
Fitting of the transition width by $d_{L}=\arctan\left(\exp(\pi L/W)\right)/\pi +1/2$ for the left region and the right region. Note that although the two are plotted together in one figure, they are fitted independently.

As shown in Figure 6, $W_{90}>W_{30}$ given the same λ, which agrees with previous results [38].

Furthermore, we find that the interface depends on the constant λ, i.e., the strength of the bottom-layer potential. This allows tuning of λ by comparing the width to the one from atomistic simulations and experimental results.

### 3.3. Atomistic Modeling

Alden and coworkers studied the width of these boundaries using scanning transmission electron microscopy (STEM). They related the width in the STEM images by comparing them to simulated STEM images based on atomic positions given by displacements of

(23) $$\Delta u=\frac{2}{\pi }\arctan\left[\exp\left(\frac{\pi x}{w}\right)\right],$$

where Δu is the fraction of the total displacement.

**Figure 7:**
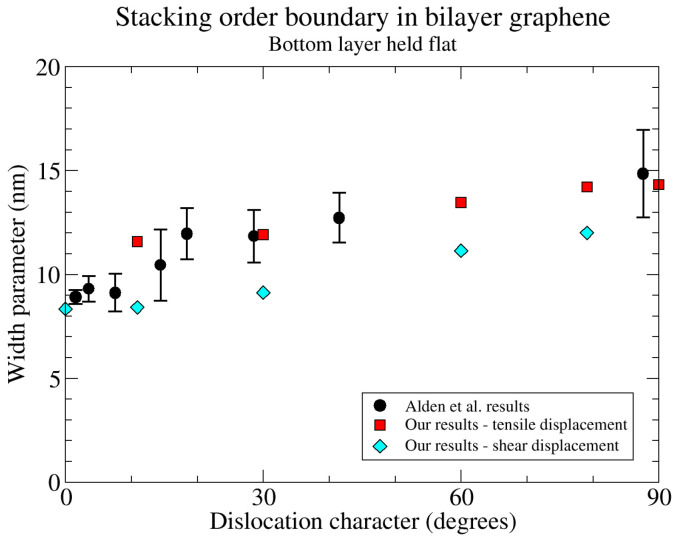
Comparison of the widths of the boundaries in our MD simulation to those in Figure 3H of reference [38]. The horizontal axis is the angle between the line of the boundary and the burgers vector of the boundary. The vertical axis is the parameter *w* in Equation (23).

Alden et al.’s primary measurements are for two graphene sheets oriented for Bernal stacking [38]. In total, there is a four-graphene-layer, free-standing sheet. The other two layers are not near to optimal stacking angles, and influence the in-plane atomic positions in the Bernal-stacking layers only weakly, but keep those sheets flat. The most relevant of our atomistic models is the one where the bottom layer is kept flat, but the atoms in that layer are allowed to relax in plane. The widths from the boundaries in those simulations agree well with Alden et al.’s results. In Figure 7, we compare the widths of the boundaries in our simulations with the widths of their experimental boundaries as shown in Figure 3H of reference [38]. The widths in their Figure 3H are full width at half-maximum (FWHM) of the STEM intensity profile. These are converted to the values *w* in Equation (23) shown in Figure 7 using the appropriate equation from the supplemental material of reference [38].

We find that Equation (23) gives an excellent fit to the displacements in both the atomistic and PFC simulations. For the flat bottom simulations, we have examined the displacement parallel to the boundary (shear displacement) and normal to the boundary (tensile displacement) separately for the mixed dislocations. We find that the Equation (23) fits the separate displacements very well, but that the widths *w* differ between the shear and tensile components. That equation also fits the component of the displacement parallel to the burgers vector, which is what we have fitted for the fixed bottom simulations for comparison with the PFC.

### 3.4. Quantify λ

**Figure 8:**
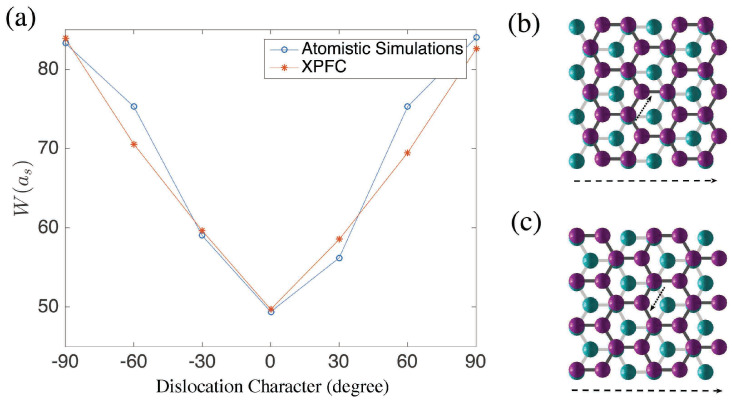
(**a**) The relation between the dislocation direction and *W* when λ=7500. The AB to BA transition enforce the atoms on the top layer (pink balls) to move along the dashed arrow. Therefore, case (**b**) is elongation (+), and case (**c**) is compression (−).

First, we find that $W_{+30}$ is different from $W_{-30}$ (this is also how we define + from −). We then investigate into this discrepancy in details and find that in the $W_{-30}$ case, for example, the distance between nearby atoms along the 30° direction is less than $a_{d}$, while in the $W_{+30}$ cases, this distance is larger. This can be explained by Figure 8. Assume that the dislocation between the top and bottom layer increases towards the right hand side. In Figure 8b, the atoms on the top layer must shift upright, denoted by the dashed arrow. The other way around is impossible because the two layers would then form an AA or BB pattern with maximum bottom-layer potential [38]. Therefore, the average distance between nearby atoms along the shifting direction will increase. For Figure 8c, the atoms on the top layer must shift bottom left in order to minimize the bottom-layer potential while the average distance between the atoms along the shifting direction will be smaller than $a_{d}$. As discussed for the MD simulations, if the bottom layer could relax, rather than being represented by $V_{\mathrm{BL}}$, this would change.

From the fixed-bottom MD simulations, we have the following data: $W_{0}=49a_{s}$, $W_{+30}=56a_{s}$, $W_{-30}=59a_{s}$, $W_{\pm 60}=75a_{s}$, $W_{+90}=84a_{s}$, and $W_{-90}=83a_{s}$, which we will use to choose a value for λ. The boundary thickness $84a_{s}$ is a comparatively large because the whole ribbon must be long enough to ensure that at least more than half of the entire domain are good stacking structures. Therefore, we use a domain of 1024×(32×1024), 32 unit blocks with a little less than half of AB and equally of BA, i.e., $L_{1}=640a_{s}$ or $L_{2}=640\sqrt{3}a_{s}$.

Starting from $\psi_{o}\left(X\right)$, we use a bisection method to pin down the value of λ, i.e., $\lambda_{0}$ = 20,000, $\lambda_{1}$ = 5000, $\lambda_{2}$ = 12,500, …. We compare the value of $W_{\lambda }^{0}$ and $W_{\lambda }^{90}$ with the atomistic results, that if the relative difference between the PFC results and atomistic results are smaller than 5% for all the cases, we then take that λ. We did not use the value of $W_{\lambda }^{\pm 30}$ or $W_{\lambda }^{\pm 60}$ since the domain is a narrow ribbon and the exact direction of the transition region is not quite precise for those two cases.

**Figure 9:**
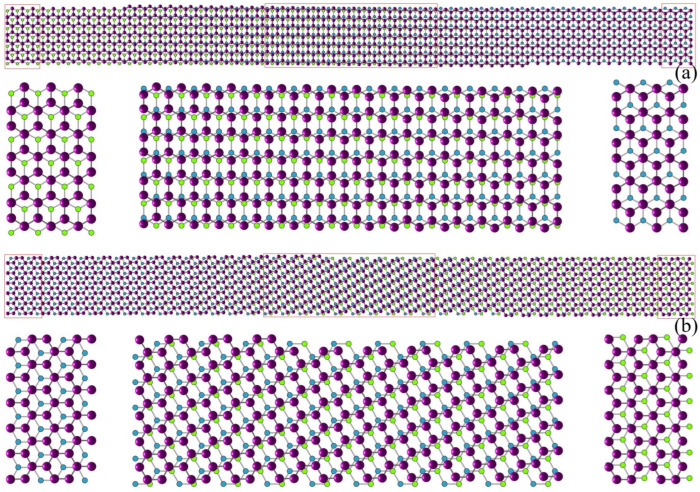
(**a**) Transition region for 0 degree. (**b**) Transition region for 30 degree.

By this procedure, we found that λ=7500 is a proper value, considering all the three cases $W_{0}$ and $W_{\pm 90}$, as shown in Figure 8. Note that for a single value of λ, our PFC results agree reasonably well with our MD simulations for all boundary characters. To illustrate the actual structure of these boundaries, in Figure 9, we plot the double layer graphene of type 0° and 30° from the PFC simulations.

### 3.5. A Circular Shape Transition Between AB and BA

**Figure 10:**
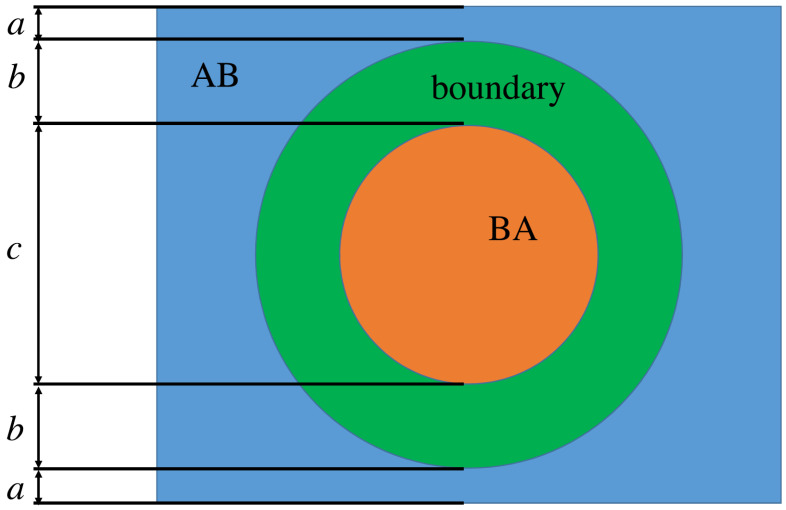
A script of the initial phase-field for the circular shape transition between AB and BA.

Another good setup to be investigated into is the dynamics of a (circular) disk-shaped grain of one stacking order, with the rest of the cell the other stacking order. One expects the circular grain to shrink and the final configuration to be a uniform stacking order. However, this is not what is observed in experiments. The initial phase-field is created following Figure 10, where in the boundary ψ=0.3 is a constant.

#### 3.5.1. Stable Inner Region with Defective Carbon Rings

**Figure 11:**
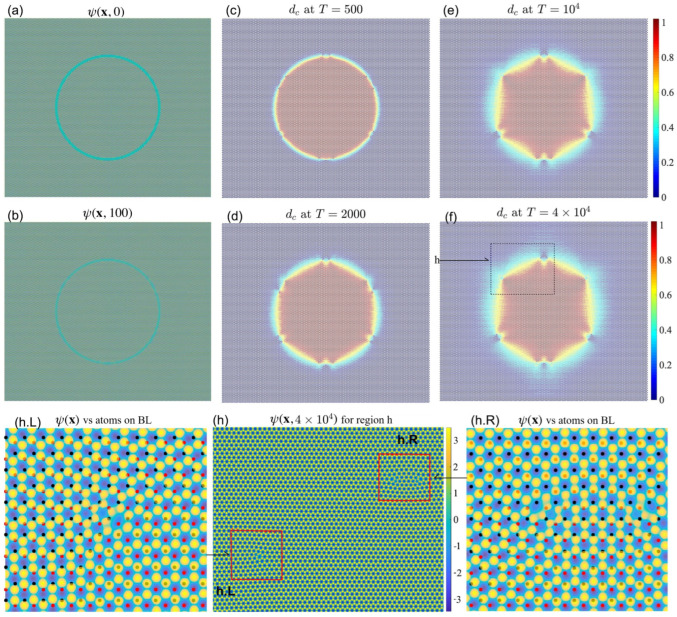
(**a**) The initial phase-field ψ(x,0). (**b**) ψ(x,100). (**c**) $d_{c}$ at T=500. (**d**) $d_{c}$ at T=2000. (**e**) $d_{c}$ at $T=10^{4}$. (**f**) $d_{c}$ at $T=4\times 10^{4}$. (**h**) Zoomed-in boxed region from (**f**). (**h.L**,**h.R**) Magnified view of the phase-field in the sub-patches. The black and red dots denote the two different sublattice sites (A and B, respectively) of the bottom graphene layer. Note that all sub figures showing the phase density ψ(x) use one same color bar (here in (**h**)), and all sub figures showing $d_{c}$ use another color bar, and below the same.

We set $a=18a_{s}$, $b=4a_{s}$, and $c=48a_{s}$, and the transition region is relatively narrow, as shown in Figure 11a. The outer region is of AB stacking order, and the interior region is of BA stacking order. Once the dynamics start, both of the AB and BA regions grow gradually, as shown in Figure 11b. They meet each other and form the transition boundary with defected points, as shown in Figure 11c. Then, the width of the transition boundary increases, as shown in Figure 11d,e. Finally, the phase-fields reach an equilibrium where the BA stacking order region is similar to a hexagon, as shown in Figure 11e,f. The direction of each sides of the hexagon region is inconsistent with the orientation of the atomic alignment on the bottom layer. Here, the boundary of the central grain becomes pinned and the defects are somewhat like the situation where the upper layer is deposited on the bottom layer, and there is nucleation and growth of stacking fault regions. The defects that involve a 5-carbon ring next to a 7-carbon ring (couple of 5 and 7 rings) are certainly seen.

In Figure 11h, we zoom in the boxed region in Figure 11f, highlighting two of the six vertices. There is one couple of 5 and 7 rings on the bottom left of the boxed region, as shown in Figure 11h.L, where we further zoom in that patch. The color map shows the phase-field. The red and black dots superimposed on the map correspond to the A and B sites of the bottom graphene layer, respectively. On the upper right patch of Figure 11h, there are two couples of 5 and 7 rings, as shown in Figure 11h.R, where we zoom in the phase-field together with the atoms on the bottom layer. Note that with 5 and 7 rings, the transition from AB to BA is sharp. Moreover, with two or three (locates at the bottom vertex) couples of 5 and 7 rings, the transition could be sharper than the one couple case, as shown in Figure 11f. This partially explains the reason why the hexagon region stagnates (the positions of the defected vertices do not change from $T=10^{4}$ to $T=4\times 10^{4}$).

We then test multiple cases of the *c* values and find that the inner hexagon region can be stable for a relatively small size, i.e., $c=24a_{s}$.

**Figure 12:**
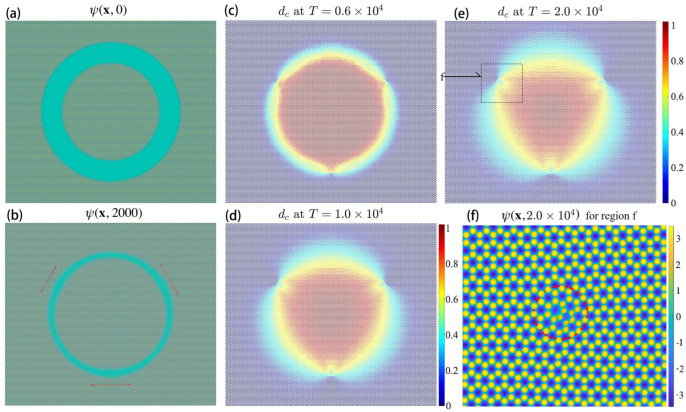
(**a**) The initial phase-field ψ(x,0). (**b**) ψ(x,2000). (**c**) $d_{c}$ at T=6000. (**d**) $d_{c}$ at $T=10^{4}$. (**e**) $d_{c}$ at $T=2\times 10^{4}$. (**f**) Zoomed-in boxed region from (**e**).

When the transition region is wider, i.e., $a=12a_{s}$, $b=16a_{s}$, and $c=36a_{s}$, the domain could evolve to a triangular shape, as shown in Figure 12. Similarly, the outer and inner regions are of AB and BA stacking order, correspondingly, as shown in Figure 12a, where the transition region is significantly wider than the case in Figure 11a. Once the dynamics start, both the AB and BA regions grow gradually towards each other and finally meet in the middle, before a structured pattern emerges independently in the transition region (this does happen if the transition region is too wide, i.e., $b=48a_{s}$), as shown in Figure 11b. On the other hand, the growing speed is not uniform along the ring. We find three parts where both the outer and inner regions grow slightly slower. Furthermore, those parts are at the positions where the defects that involve one couple of 5 and 7 rings finally emerge. Also note that at those positions, the direction of the transition region (depicted by red arrows) is parallel to the direction of nearby empty holes in the phase-field, as shown in Figure 11f.

As the AB and BA regions grow, they will meet each other and form the transition boundary with defected points, as shown in Figure 12c. Then, the width of the transition boundary increases, as shown in Figure 11d. Finally, the simulation reaches an equilibrium where the BA stacking order region is of triangular shape, as shown in Figure 12e. Similarly as the previous case, the defects that involve a couple of 5 and 7 rings are certainly seen, as shown in Figure 12f, where we zoom in on the boxed patch from Figure 12e.

#### 3.5.2. Shrinking Inner Region with Uniform Transition

Finally we simulate a case that the center grain does shrink and finally goes away. The initial condition is specifically generated that the direction of the shifting displacement in the transition region is vertical uniformly. We use a linear transition for simplicity. Suppose $d_{c}=0$ for the outer structure, and $d_{c}=1$ for the inner structure. Let *r* be the distance from x to the center of the box, $R_{i}$ the inner radius of the boundary region, and $R_{o}$ the outer radius of the boundary region. In the phase-field, we set $d_{c}\left(x\right)$ by

(24) $$\begin{array}{cc} \mathrm{if}\;r>R_{o}, & \;\mathrm{then}\;d_{c}\left(x\right)=0.\\ \mathrm{if}\;r<R_{i}, & \;\mathrm{then}\;d_{c}\left(x\right)=1.\\ \mathrm{if}\;R_{i}<r<R_{o}, & \;\mathrm{then}\;a=\left(R_{o}-r\right)/\left(R_{o}-R_{i}\right),\;\mathrm{and}\;d_{c}\left(x\right)=1-a. \end{array}$$

**Figure 13:**
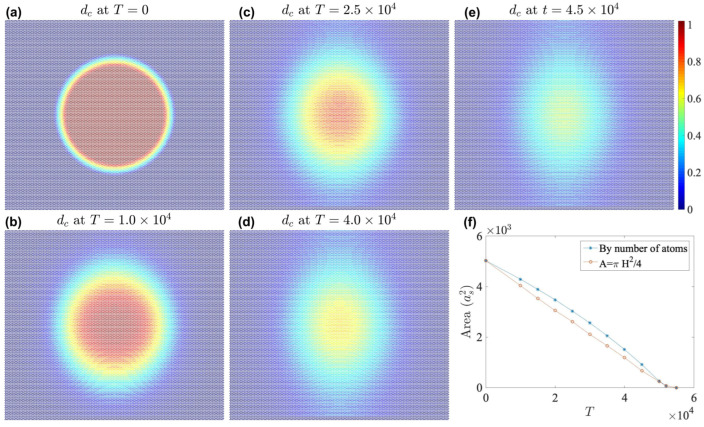
(**a**) $d_{c}$ at T=0. (**b**) $d_{c}$ at $T=10^{4}$. (**c**) $d_{c}$ at $T=2.5\times 10^{4}$. (**d**) $d_{c}$ at $T=4.0\times 10^{4}$. (**e**) $d_{c}$ at $T=4.5\times 10^{4}$. (**f**) The area of the central BA region as a function of time. All figures showing $d_{c}$ use one identical color bar.

In Figure 13, we present a sample case, with $R_{i}=42a_{s}$ and $R_{o}=50a_{s}$. Figure 13a shows the initial condition. Note that it takes a short period for the phase domain to evolve to a state with a ’natural’ transition region; thus, we only use data points after $T=10^{4}$. As shown by Figure 13b–e, the center keeps shrinking. On the other hand, the center grain does not stay circular as it shrinks. In Figure 13c–e, the BA region is elliptical, and its aspect ratio grows. This actually agrees with previous results: the transition width *W* for θ=0° is the smallest, i.e., the left and right parts of the central ellipse. *W* increases as θ increases, and reaches the maximum when θ=±90°, i.e., the top and bottom parts.

In Figure 13f, we plot the area of the central grain as a function of time. Here, we use two methods to measure the area. By the first method, we simply count the numbers of atoms with $d_{c}>0.5$, that each atoms occupies an area of $\sqrt{3}a_{s}^{2}/4$. Since only one group of atoms on the bottom layer are counted, we multiply the value by 2 to represent the total area for convienence. By the second method, we measure the width *H* from the left vertex to the right vertex of the central region, i.e., the length of the short axis of the ellipse. As shown in Figure 13f, for the first case, the area shrinks slower at the beginning and then faster. For the second case, the area as a function of time is linear, which is the expected behavior if the boundary motion is governed by a constant mobility. It could be a good sign for the possibility of using the method for at least qualitative dynamics.

We further test the case that $R_{i}=88a_{s}$ and $R_{o}=96a_{s}$ and find that the center grain still shrinks, although it shrinks much more slowly than the above case because of the lower curvature of the boundary.

## 4. Conclusions

In this work, we have developed a phase-field crystal model tailored for bilayer graphene by extending the structural PFC methodology. A central feature of our model is the incorporation of an external potential, derived from first-principles calculations of the generalized stacking fault energy, to represent the influence of a fixed bottom graphene layer. This approach allows the PFC free energy functional to accurately capture the energetics of different stacking orders and the transitions between them.

One accomplishment of this study is the quantitative calibration of the model parameter, λ, which scales the strength of the interlayer interaction potential. By simulating the width of the transition regions (stacking fault boundaries) between AB and BA domains for various dislocation characters and comparing these results with molecular dynamics simulations, we determined an optimal value of λ=7500. This calibration ensures that our PFC model reproduces interface widths consistent with atomistic simulations, thereby bridging the gap between the coarse-grained PFC description and more detailed atomic-scale methods.

Our simulations successfully demonstrate the model’s capability to capture complex microstructural evolution in bilayer graphene. Notably, we observed that an initially circular domain of one stacking order embedded in the other can evolve into stable, non-circular shapes (triangular or hexagonal) bounded by dislocation networks featuring 5–7 carbon rings. The stability of these shapes is attributed to the pinning of the boundary at these defect vertices. Conversely, when initialized with a smooth, uniform transition, the central domain shrinks at a rate governed by boundary curvature, indicating the model’s potential for studying domain growth kinetics.

In summary, this work establishes a quantitatively calibrated phase-field crystal framework for investigating stacking phenomena and defect dynamics in bilayer graphene. The model efficiently simulates processes at diffusive time scales that are challenging for atomistic methods, while retaining essential atomic-level structural information. This makes it a valuable tool for exploring microstructure evolution in bilayer graphene systems, with potential applications in understanding defect engineering and stability in twisted bilayers or other two-dimensional heterostructures.

## Data Availability

The data presented in this study are available on request from the corresponding author.
